# Hepatic Arterial Infusion Chemotherapy in the Treatment of Unresectable Hepatocellular Carcinoma with and Without Extrahepatic Spread: A Propensity Score Matching Study

**DOI:** 10.3390/jpm15110561

**Published:** 2025-11-19

**Authors:** Chao-Ting Chen, Huei-Lung Liang, Chia-Ling Chiang, Wei-Lun Tsai, Yu-Chia Chen

**Affiliations:** 1 Department of Radiology, Kaohsiung Veterans General Hospital, Kaohsiung 813414, Taiwan; ctchen@vghks.gov.tw (C.-T.C.); clchiang1104@vghks.gov.tw (C.-L.C.); 2 Interventional Center, Antai Tian-Sheng Memorial Hospital, Pingtung County 928004, Taiwan; 3 Division of Gastroenterology and Hepatology, Department of Medicine, Kaohsiung Veterans General Hospital, Kaohsiung 813414, Taiwan; wltsai@vghks.gov.tw; 4 Department of Medical Imaging and Radiology, Shu-Zen Junior College of Medicine and Management, Kaohsiung 813414, Taiwan; 5 Department of Surgery, Kaohsiung Veterans General Hospital, Kaohsiung 813414, Taiwan; ycchen@vghks.gov.tw

**Keywords:** hepatocellular carcinoma, hepatic arterial infusion chemotherapy, extrahepatic spread, lipiodol, microvascular embolization

## Abstract

**Purpose:** We aimed to study the efficacy and safety of hepatic artery infusion chemotherapy (HAIC) in the treatment of unresectable hepatocellular carcinoma (HCC) with extrahepatic spread (EHS). **Materials and Methods:** A total of 323 patients with unresectable HCC received HAIC plus lipiodol microvascular embolization. HAIC was performed via puncture of the left subclavian artery with a temporary 4-French angio-catheter placed in the common/proper hepatic artery. The HAIC regimen consisted of a daily infusion of cisplatin (10 mg/m^2^), mitomycin-C (2 mg/m^2^), and leucovorin (15 mg/m^2^), administered over a period of 20–30 min, and then a 5-fluorouracil (5-FU, 100 mg/m^2^) infusion for the remaining of 22 h of each day, for five consecutive days. Before the temporary catheter was removed, 10 mL of ethiodized oil (Lipiodol, Guerbet, France) was injected to obtain a synergistic effect of chemoinfusion and lipiodol microvascular embolization. Treatment responses were evaluated based on mRECIST criteria. The objective response rate (ORR), progression-free survival (PFS), and overall survival (OS) of patients with EHS were compared to those without. Subgroup analyses of patients with and without major portal vein tumor thrombosis (PVTT) were performed both before and after propensity score matching (PSM). The survival analyses were conducted with the Kaplan–Meier method and compared using the log-rank test. All the statistical analyses were performed by SPSS (version 26.0). **Result:** The overall ORR was 59.1%. The median OS of the initial cohort and patients positive and negative for EHS were 16.3, 12.0, and 18.0 months, respectively (*p* = 0.002). In the subgroup analysis, there was no statistical difference in survival in patients with major PVTT between the with-EHS and without-EHS groups (13.0 vs. 15.0 months, *p* = 0.407). However, the median OS in patients with EHS was significantly shorter than those without EHS (11.4 vs. 19.4 months, *p* < 0.001) in the subgroup of non-major PVTT patients. After PSM, there were no significant survival differences between the EHS and non-EHS groups in any patient cohort or sub-cohort analysis. **Conclusions:** For unresectable HCC, controlling intrahepatic tumor progression through HAIC is more important than controlling extrahepatic tumor growth, especially in patients with major PVTT. Personalized locoregional HAIC can be performed in patients with EHS.

## 1. Introduction

Hepatocellular carcinomas (HCCs) with major portal vein tumor thrombosis (PVTT) or extrahepatic spread (EHS) are classified as Barcelona Clinic Liver Cancer (BCLC) stage C or as “advanced” HCCs [1]. For this patient population, despite marginal clinical benefits, sorafenib has remained the main treatment option for over a decade [2,3,4]. The overall survival (OS) and/or hazard ratio (HR) of HCC patients with EHS were reported to have no statistically significant differences versus those without EHS (11 versus 9.6 months, *p* = 0.765) for those who received sorafenib [5].

Hepatic artery infusion chemotherapy (HAIC) is an effective treatment for advanced HCC [6,7,8,9]. Zhuang’s meta-analysis showed a positive objective response rate (ORR; odds ratio, 0.13; 95% confidence interval (CI), 0.07–0.24) and disease control rate (odds ratio 0.48; 95% CI, 0.26–0.87) for HAIC when compared to sorafenib [6]. Another meta-analysis by Liu found better OS and progression-free survival (PFS) for the HAIC group compared to the sorafenib group in HCC with PVTT [7]. Other studies have shown that combining HAIC with sorafenib resulted in better OS rates [8,9,10,11].

There are significant differences in nature among the population of patients with advanced HCC. Whether such patients have also PVTT and EHS are clinical factors influencing survival. Up to 64.7% of HCC cases are accompanied by PVTT [12]. Previous research and Japanese guidelines suggest that HAIC can be used as a treatment option for HCC with PVTT [13]. However, there is some controversy regarding whether HAIC is effective for patients with EHS, mainly because HAIC is considered a locoregional therapy and may have limited disease control effects. Previous studies have yielded conflicting results regarding the effects of EHS on the survival impact of HAIC. Kim et al. [14] reported an OS of 7.6 months without significant survival difference in HCC patients with or without EHS (HR: 1.101, *p* = 0.63). Two studies [15,16] found that EHS results in poorer OS (7.7 vs. 9.8 months and 9.8 vs. 14.8 months, respectively) without reaching statistical significance (*p* = 0.068); however, two other studies showed that EHS was an independent factor for OS, with HRs of 1.76, *p* = 0.0011, [10] and 1.71, *p* = 0.01 [17], respectively. The conflicting results of the locoregional treatment effects for EHS patients are most likely due to different HAIC regimens adopted, as controlling intrahepatic tumor progression is more crucial than controlling that of extrahepatic growth. The latter two studies with significance had adopted either a new FP regimen [10] or new FOLFOX regimen [17] that had been reported to be more effective in controlling the intrahepatic tumor progression.

Nowadays, systemic immune-target therapy (atezolizumab plus bevacizumab) has become the mainstay to treat advanced HCC patients. Guan et al. [18] and Chen et al. [19] reported that the median OS rates of immune-target therapy alone in EHS patients were 9.0 and 8.4 months, respectively, which are still suboptimal. Thereafter, a new treatment strategy with either a new HAIC regimen and/or combination therapy is warranted.

We had previously reported a new potent HAIC regimen combining conventional HAIC and lipiodol microvascular embolization in treating advanced HCC patients with main portal vein invasion, with encouraging clinical outcomes [11]. But the rationale and treatment outcomes when treating advanced HCC patients with EHS is still unclear. Therefore, in this study, we retrospectively reviewed 323 unresectable HCC patients with and without EHS treated with our new HAIC regimen. The clinical outcomes and risk factors for both groups were analyzed using before and after propensity score matching (PSM). The primary objective of this study is to evaluate the precision medicine efficacy and safety of HAIC in treating unresectable HCC patients with EHS.

## 2. Method

**Figure 1:**
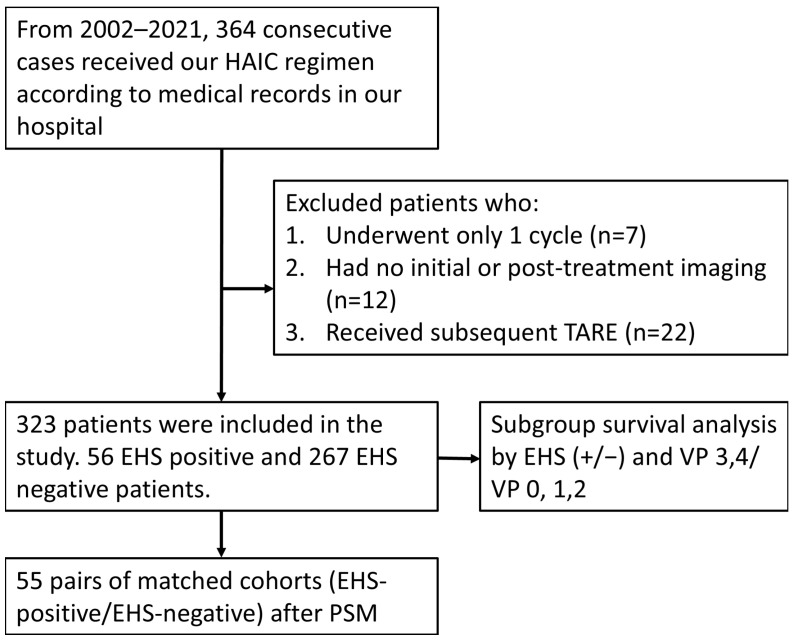
Flow diagram for study participants and exclusion criteria.

This was a retrospective large cohort study in one single institute, with a total of 323 consecutive unresectable HCC patients enrolled at Kaohsiung Veterans General Hospital from April 2002 to December 2021. HCC diagnosis was based on imaging with proper clinical context or pathology confirmation. The inclusion criteria were as follows: (a) patients aged 18 years old or older; (b) Child–Pugh class A and B; (c) platelet counts ≥ 50,000/cumm; (d) prothrombin time INR ≤ 1.5; (e) tumor mass ≥ 8 cm or with major PVTT or EHS; (f) having received at least two courses of HAIC; (g) ECOG performance status ≤ 2. The exclusion criteria were as follows: (a) patients who subsequently underwent transarterial radioembolization (TARE); (b) patients who did not have initial or post-treatment imaging available on our PACS system (Figure 1). Patients’ follow-ups were performed by reviewing medical records from the PACS system of our hospital, with a mean follow-up period of 26.0 ± 31.7 months. The follow-up period ended in December 2023 This study was approved by the ethics review committee of Kaohsiung Veterans General Hospital, with patient informed consent waived for this retrospective study.

The severity of PVTT was categorized according to the Japanese VP Staging Classification System [20], where Vp4 was defined as the presence of a tumor thrombus in the main trunk of the portal vein or a portal vein branch contralateral to the primarily involved lobe; Vp3 and Vp2 as tumor invasion of the first-order and second-order branches, respectively; and Vp1 and Vp0 as no visible vascular invasion on images.

HAIC was performed via puncture of the left subclavian artery [21] under ultrasound guidance with insertion of a 4-French temporary angio-catheter (RC1, Cordis, Miami Lakes, FL, USA; J-curve, Terumo, Tokyo, Japan) in the common hepatic artery or in the proper hepatic artery. The gastroduodenal artery and/or right gastric artery were embolized with metallic coils during the first HAIC therapy to prevent non-target gastrointestinal injury. Our new HAIC regimen consisted of a daily infusion of cisplatin (10 mg/m^2^), mitomycin-C (2 mg/m^2^), and leucovorin (15 mg/m^2^) administered over a period of 20–30 min separately, and then a 5-fluorouracil (5-FU, 100 mg/m^2^) infusion for the remaining 22 h of each day, for five consecutive days. Before the temporary catheter was removed, 10 mL of ethiodized oil (Lipiodol, Guerbet, France) was injected into the hepatic arteries via the already-placed angio-catheter to obtain a synergistic effect of chemoinfusion and lipiodol microvascular embolization. In patients with near-complete response, severe arterioportal shunt causing high lipiodol reflux, or shunting to the contralateral lobe, a reduced lipiodol amount (5 mL) was injected. If tumor(s) had two major blood supplies from both the celiac trunk and the SMA, the chemo-agents and lipiodol dose was divided into two halves and given for 2.5 days each, sequentially.

Neither gelfoam nor any other embolizing particles were injected to prevent potential irreversible liver damage. No permanent delivery port system was used in this study. Adjunctive therapies such as surgical resection, percutaneous ablation, transcatheter arterial chemoembolization (TACE), and radiotherapy were performed in partial-response patients after at most 6 courses of HAIC at clinicians’ and patients’ discretion.

As systemic sorafenib target therapy was reimbursed in 2010 in Taiwan and available in the study hospital after 2012, combination therapy of HAIC and sorafenib (400 mg, bid) concurrently was performed in advanced HCC patients (mPVTT and/or EHS) with Child–Pugh A liver function after 2012.

The interval between each course of the five-day treatment was 6–8 weeks. Multiphasic CT or MRI evaluation for treatment response was performed after every two courses of treatment. As HAIC is a locoregional therapy, intrahepatic tumor response was adopted to judge treatment efficacy instead of disease response. Treatment efficacy was therefore classified into complete response (CR), partial response (PR), stable disease (SD), and progressive disease (PD) according to the Modified Response Evaluation Criteria in Solid Tumors (mRECIST) guidelines [22]. The interpretation of imaging response of the 323 advanced HCC patients was allocated to 3 interventionalists (Chen C.T., Liang H.L., and Chiang C.L.) with 4–30 years’ experiences of abdominal imaging diagnosis. As the primary goal of this study was to evaluate the HAIC therapeutic outcomes (PFS and OS), we did not ensure inter-observer consistency in interpreting intrahepatic tumor response.

Propensity score matching (PSM) analysis with one-to-one matching was used to balance baseline characteristics between groups with and without EHS, as well as groups with and without combined sorafenib use. The propensity scores were estimated using a logistic regression model that included the following variables: gender, age, HCC etiology, EHS, maximal tumor size, tumor number, major PVTT, Child–Pugh class, and AFP level. Nearest-neighbor matching without replacement was applied, with a caliper set at 0.02.

The Kaplan–Meier method was used to compare progression-free survival (PFS) and overall survival (OS) between groups, and the log-rank test was used for statistical comparison. A *p*-value of <0.05 was considered statistically significant. Multivariate analysis using a Cox proportional hazards model was performed to identify clinical factors influencing overall survival. Variables that achieved statistical significance or had a *p*-value < 0.10 in univariate analysis were included in the multivariate analysis. A *p*-value < 0.05 was considered statistically significant in the multivariate analysis. The Statistical Package for the Social Sciences (SPSS, version 26.0, Inc., Chicago, IL, USA) was used for the statistical analyses.

## 3. Result

Table 1 presents the baseline characteristics of all the enrolled patients both before and after PSM. Before matching, the mean age was 60.9 ± 11.4 years, with 82.7% of the patients being male. A total of 84.2% of the patients had underlying viral hepatitis (B or C virus infection), 162 patients (50%) had a tumor size larger than 8 cm, 55 patients (17%) had tumor numbers greater than 10, 51% of patients had alpha-fetal protein (AFP) levels higher than 400 ng/mL, and 86.4% were classified as Child–Pugh class A when HAIC treatment began.

Regarding PVTT severity, 25.1% of patients had VP4, 24.1% had VP3, 28.8% had Vp2, and 22% showed either Vp0 or Vp1 involvement. Among all enrolled patients, a total of 56 (17.3%) individuals exhibited extrahepatic metastasis (EHS) at the time of enrollment. Of these patients, lung, adrenal gland, and regional lymph node metastasis were seen in 19, 5, and 37 patients, respectively. Therefore, 265 (82.0%) patients were classified as BCLC-C and 58 (18.0%) as BCLC-B in this study.

**Figure 2:**
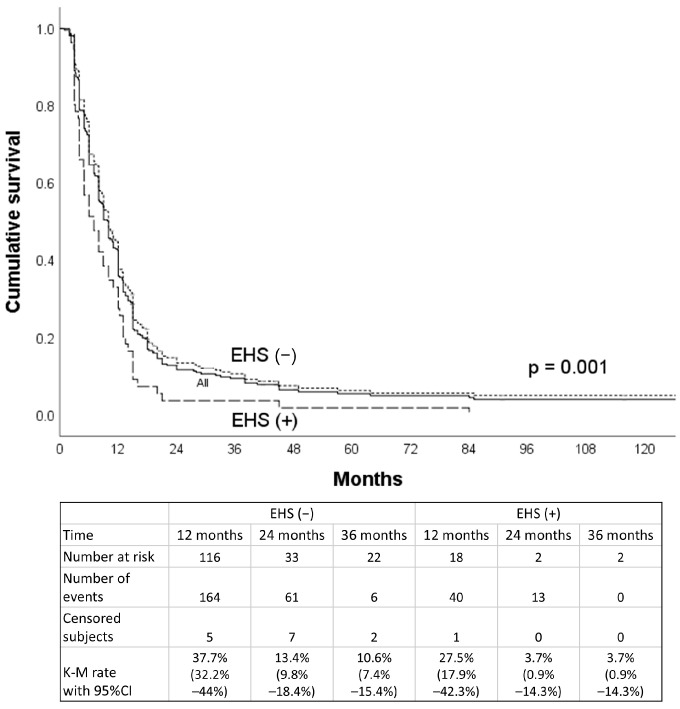
Progression-free survival curves of the whole cohort and subgroups with and without EHS before propensity score matching.

The median and mean number of HAIC treatment cycles completed by each patient was 4 and 4.1 ± 1.8. A total of 270 of the 323 patients (83.6%) died by 15 June 15 2023. The intrahepatic treatment response—categorized as all patients, patients without EHS, and patients with EHS—was evaluated using the mRECIST criteria after at least two cycles of HAIC. In total, 13.9% of all patients, 15.4% without EHS, and 7.1% with EHS achieved intrahepatic CR; 45.2% of all patients, 45.7% without EHS, and 42.9% with EHS showed intrahepatic PR; 22.3% of all patients, 22.5% without EHS, and 21.4% with EHS experienced SD; and 18.6% of all patients, 16.5% without EHS, and 28.6% with EHS demonstrated intrahepatic PD, respectively. The objective response rate (ORR) was 59.1% of all patients, 61.1% without EHS, and 50% with EHS, respectively. The median PFS was 10.0, 10.0, and 7.0 months, respectively (Figure 2). The 12-month PFS was significantly different between the groups of patients without EHS and with EHS (37.7% vs. 27.5%, *p* = 0.001).

**Figure 3:**
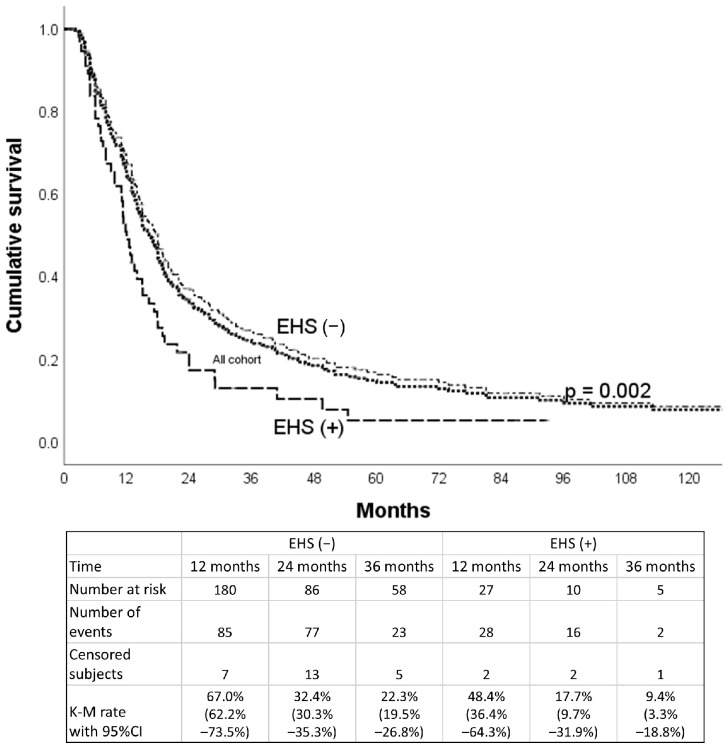
Overall survival curves of the whole cohort and subgroups of patients with and without EHS before propensity score matching.

**Figure 4:**
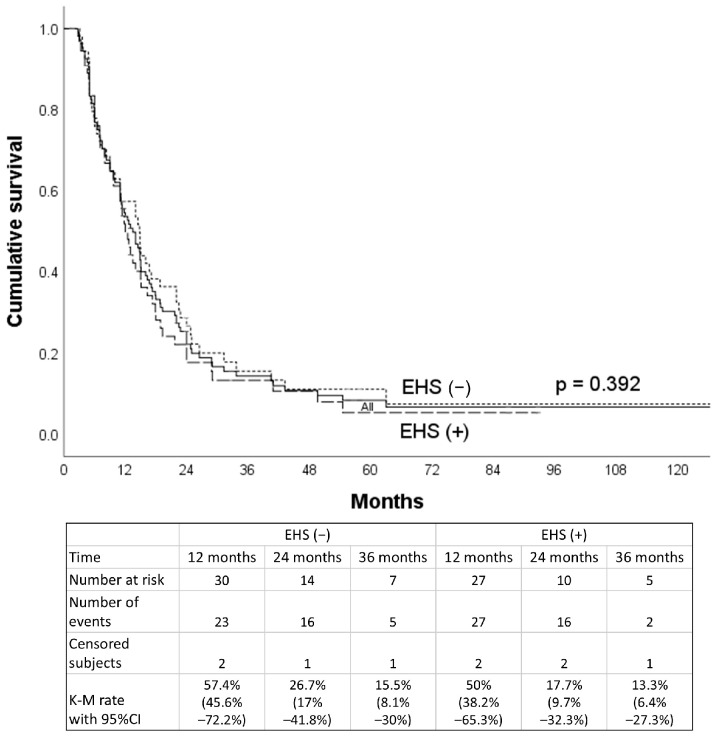
Overall survival curves of the whole cohort and subgroups of patients with and without EHS after propensity score matching.

The median OS for all patients was 16.3 months (95% CI, 14.5–18.1), compared to 18.0 months for the non-EHS patients and 12.0 months for the EHS patients. The 1-, 2-, and 3-year OS rates were, respectively, 67.0% (95% CI, 62.2–73.5%), 32.4% (95% CI, 30.3–35.3%), and 22.3% (95% CI, 19.5–26.8%) for the non-EHS group and 48.4% (95% CI, 36.4–64.3%), 17.7% (95% CI, 9.7–31.9%), and 9.4% (95% CI, 8.3–18.8%) for the EHS patients (*p* = 0.002). After PSM (55 pairs), the median OS was 12.0 months (95% CI, 10.0–14.0 months) for the EHS-positive group (Figure 3) and 14.8 months (95% CI, 10.8–18.8 months) for the EHS-negative group (Figure 4). The 1-, 2-, and 3-year OS rates were, respectively, 57.4% (95% CI, 45.6–72.2%), 26.7% (95% CI, 17.0–41.8%), and 15.5% (95% CI, 8.1–30.0%) for the non-EHS group and 50.0% (95% CI, 38.2–65.3%), 17.7% (95% CI, 9.7–32.3%), and 13.3% (95% CI, 6.4–27.3%) for the EHS patients. There was no significant difference in survival between the EHS and non-EHS groups (*p* = 0.392). For responsive patients (CR + PR), the median OS of the entire initial cohort was 24 months (95% CI, 20.0–28.0 months), which was significantly longer (*p* < 0.0001) than that of the non-responders (SD + PD), at 8.3 months (95% CI, 7.4–9.2 months).

**Figure 5:**
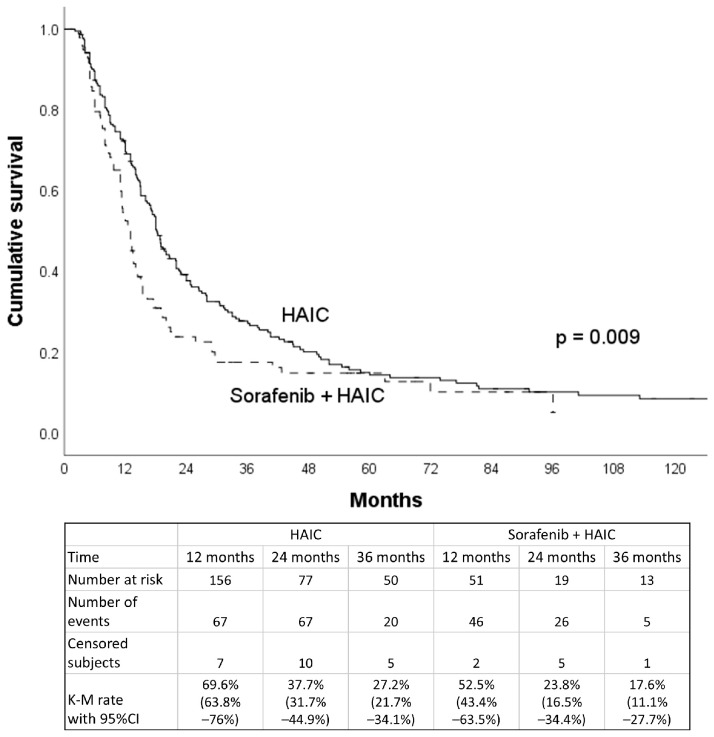
Overall survival curves for patients receiving HAIC treatment or receiving HAIC combined with sorafenib before propensity score matching.

**Figure 6:**
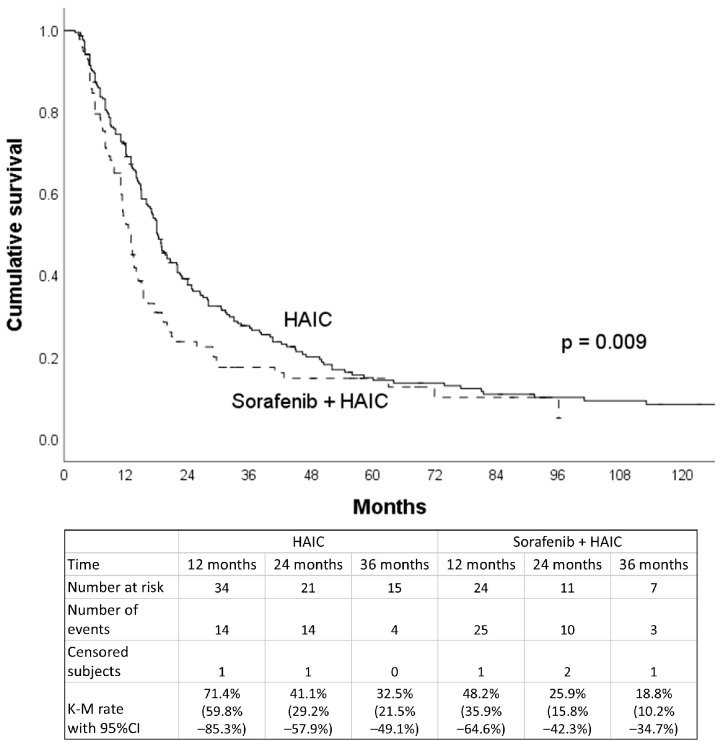
Overall survival curves for patients receiving HAIC treatment or receiving HAIC combined with sorafenib after propensity score matching.

Subgroup analysis showed the median OS for the 159 HCC patients with major PVTT was 13.0 months in patients with EHS (95% CI, 9.2–16.8 months) and 15.0 months for those without EHS (95% CI, 12.2–17.8 months). There was no significant difference in survival when comparing EHS and non-EHS patients in the major PVTT group (*p* = 0.407). For the 164 patients without major PVTT, the median OS was 18.0 months, with the 11.4 months for patients with EHS (95% CI, 10.6–12.2 months) being significantly shorter (*p* < 0.001) than the 19.4 months for those without EHS (95% CI, 16.8–22.0 months) (Table 2). Out of the 323 patients included in the study, 98 patients (30.3%) received both HAIC and sorafenib treatment concurrently. There was a survival difference between patients who received HAIC treatment alone and those who received HAIC in combination with sorafenib (18.2 vs. 13.0 months, *p* = 0.009) (Figure 5). After PSM (49 pairs) adjusting for gender, age, HCC etiology, EHS, maximal tumor size (≥8 cm), tumor number (≥10), major PVTT, Child–Pugh class, and AFP level (≥400 ng/mL), there was no survival difference between these two groups (18.8 vs. 12.0 months, *p* = 0.314) (Figure 6). As for extrahepatic metastatic sites, there was no significant difference in the median OS between HCC patients with lung and non-lung metastasis (11.3 vs. 13 months, *p* = 0.370).

**Figure 7:**
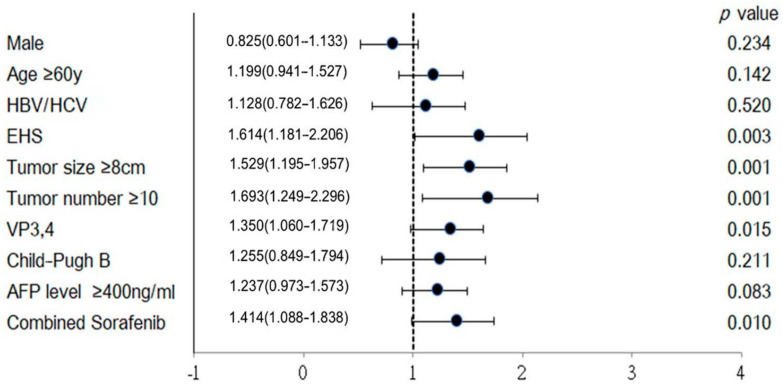
Hazard ratios of survival in univariate analysis.

Univariate analysis of the prognostic factors associated with survival is shown in Figure 7. Positive EHS, maximal tumor size ≥8 cm, tumor number ≥ 10, major PVTT, and AFP level ≥ 400 ng/mL were significant risk factors. Under multivariate analysis, tumor number ≥ 10 (HR 1.505; 95% CI, 1.101–2.058; *p* = 0.010) and major PVTT (HR 1.298; 95% CI, 1.013–1.663; *p* = 0.039) were significant risk factors. (Figure 7)

As for the adverse events of this treatment, none of the patients died due to immediate HAIC complications. Sixty four patients developed fever during the HAIC period, and among them, twelve developed bacteremia and were treated successfully by antibiotics. Transient ischemic attacks after catheter removal were encountered in two patients, with spontaneous recovery within 3 days. One patient showed pseudoaneurysm formation in the left punctured subclavian artery, which was treated successfully with a stent graft (Viabahn, 7 × 50 mm, Gore), placed via the left brachial approach. Three patients developed an overt subcutaneous hematoma at the puncture site requiring no further management. No overt adverse events were observed in patients receiving HAIC plus sorafenib therapy.

## 4. Discussion

The present study revealed that the presence of EHS affected overall survival (12.0 vs. 18.0 months, *p* = 0.002) throughout the entire cohort of patients with unresectable HCC who received HAIC, which was consistent with the conclusions from Iwamoto’s [10] and Liang’s studies [17]. They both concluded that the presences of EHS and/or major PVTT were independent factors in multivariable analysis, whereas our subgroup analysis further indicated that although there was a significant survival difference in ≤Vp2 HCC patients between those with and without EHS (11.4 vs. 19.4 months, respectively, *p* < 0.001), there was no significant survival difference in HCC patients with major PVTT (13.0 months with EHS vs. 15.0 months without, *p* = 0.407).

These results seem reasonable since the OS of HCC patients with major PVTT has poor prognoses of 2.7–4 months under the best supportive care [23,24] and 7.6 months with immune-target combination therapy of atezolizumab plus bevacizumab in Vp4 patients [25]. HCC patients with major PVTT are also reported in the literature to have a high percentage of association with EHS (48.3–83.8%) [12,13,14,15,16]. In the present study, 57.1% of HCC patients with major PVTT had EHS. Previous studies suggested that intrahepatic tumor progression was the main cause of advanced HCC-related deaths. If intrahepatic HCC responded to HAIC treatment, this could significantly increase overall survival [13,25], but if the ORRs of the intrahepatic tumors were low (e.g., <30%), then a shorter OS was inevitable with or without EHS, and no survival difference between these two groups of patients could be expected [10,14,15,16,17]. Although EHS was one of the risk factors for OS in the univariate analysis in the present study, only tumor number (>10) and major PVTT were prognostic factors in the multivariate analysis. Controlling intrahepatic tumors through HAIC may be more important than controlling extrahepatic tumors, especially in HCC patients with major PVTT. Therefore, adopting an effective HAIC regimen of precision medicine to control intrahepatic tumor progress is absolutely crucial.

The treatment protocol used in the present study combined HAIC and lipiodol-only microvascular embolization after a 5-day chemoinfusion to obtain a synergistic therapeutic effect. Overall, the study patients had an ORR of 59.1%, with CR, PR, SD, and PD rates of 13.9%, 45.2%, 22.3%, and 18.6%, respectively. In addition, the median OS for patients with major PVTT was 14.9 months. Two studies also reported using combination therapies of HAIC with lipiodol injection but sequenced in reverse [10,26]; in both studies, the mixture of anticancer drug (cisplatin or epirubicin) and lipiodol was injected at the beginning of the treatment and was then followed by continuous chemoinfusion (5 days for 5-FU in Iwamoto’s study [10] and one day of oxaliplatin plus leucovorin and 5-FU in Liu’s study [26]). Iwamoto and Liu, respectively, reported median OS values of 13 and 10 months in patients with major PVTT. It seems that the additional lipiodol injection may have enhanced the chemo-cytotoxic effect in HAIC treatment, and longer durations of continuous chemoinfusion (e.g., 5 days) may have obtained a better ORR and OS.

Our present study revealed a significant survival difference in ≤Vp2 HCC patients between those with and without EHS (11.4 vs. 18 months), that may indicate precision medicine of aggressive treatment of the EHS in these patient group of clinical benefits. Dong et al. reported that a combination therapy of HAIC with atezolizumab plus bevacizumab had significantly prolonged the OS of advanced HCC patients (around 50% patients with EHS) than that of immune-target therapy alone (14.6 vs. 11.3 months, *p* = 0.032) [27]. In the present study, the median OS of patients with intrahepatic treatment response (CR plus PR) was 24 months, which was significantly longer than those without intrahepatic response (SD plus PD, 8.3 months). This finding further confirmed the previous statement that controlling intrahepatic tumor progression is more important than that of extrahepatic spread in prolonging patients’ survival. Meanwhile the median OS of the treatment non-responders in our study was similar to that of EHS patients who received immune-target therapy alone in two recent studies reported in the literature [18,19]. Guan et al. [18] and Chen et al. [19] reported that HAIC plus immune-target therapy significantly extended the median OS in EHS patients, with median OS rates of 27 and 26 months vs. 9.0 and 8.4 months, respectively, in the groups receiving immune-target therapy alone (*p* < 0.001). The poorer survival of the immune-target therapy alone groups in the above two studies may explain the reason why there was no survival difference between the two groups of patients with and without concurrent use of sorafenib in our present study. These results may also indicate that while immune-target therapy alone is effective in suppressing EHS, it may be less effective in controlling intrahepatic tumor progression in these advanced HCC patients, and our new HAIC regimen is effective for this purpose. Therefore, it is possible that personalized medicine of the combination of our new HAIC regimen with immune-target therapy and/or other treatment modalities (e.g., proton beam therapy) for EHS lesions may prolong patients’ survivals, especially in patients with ≤Vp2 HCC. But the high medical cost of such a combination therapy should also be taken into consideration in general clinical practice.

In the present study, no significant survival difference was found between the EHS-positive and EHS-negative patient groups (*p* = 0.392), nor between those with and without sorafenib use (*p* = 0.314) after PSM adjustment. A possible reason for this is that the percentage of major PVTT patients increased in all patient groups after PSM, and major PVTT was identified as an independent risk factor (HR: 1.298) for patient survival, while EHS was a risk factor in univariate analysis, but not multivariate.

## 5. Limitations

The present study had some limitations. It was a retrospective study, so the included patients were highly heterogeneous and may have had selection bias. Additionally, there were no control groups for comparison with other treatment methods. As patients who did not receive two courses of HAIC treatment or post-treatment imaging follow-up were excluded, OS may have been skewed in a favorable direction. In the future, a large prospective study is needed to accurately control for the characteristics of enrolled patients in order to identify the patient population suitable for HAIC treatment. In addition, even though the study was adjusted by PSM, it cannot account for unmeasured variables.

## 6. Conclusions

The results of this study on precision medicine suggest that controlling intrahepatic tumors through HAIC is more important than controlling extrahepatic tumors, especially in HCC patients with major PVTT. In addition, locoregional HAIC can be performed in advanced HCC patients with EHS.

## Figures and Tables

**Table 1:** Baseline characteristics of groups with and without EHS before and after 1:1 propensity score matching (PSM).

EHS (Yes/No)	Before PSM *n* = 323			After PSM *n* = 110					
	Yes	No	*p*-Value	Yes		No		*p*-Value	SMD
Gender (M/F)	49/7	218/49	0.338	48/7	87.3 ± 4.5%	51/4	92.7 ± 3.5%	0.527	0.183
Age (≥/<60)	26/30	147/120	0.243	25/30	45.5 ± 6.7%	23/32	41.8 ± 6.7%	0.848	0.073
HBV or HCV/Others	47/9	232/35	0.526	47/8	85.5 ± 4.8%	50/5	30.9 ± 3.9%	0.556	0.168
Tumor size (≥/<8) cm	41/15	121/146	<0.001	41/14	74.5 ± 5.9%	41/14	74.5 ± 5.9%	>0.999	<0.001
Tumor number (≥/<10)	14/42	41/226	0.116	14/41	25.5 ± 5.9%	12/43	21.8 ± 5.6%	0.823	0.085
VP3,4/VP 0,1,2	32/24	127/140	0.24	32/23	58.2 ± 6.7%	33/22	60.0 ± 6.6%	>0.999	0.037
Child–Pugh A/B	49/7	230/37	>0.999	48/7	87.3 ± 4.5%	48/7	87.3 ± 4.5%	>0.999	<0.001
AFP (≥/<400) ng/mL	37/19	127/170	0.013	36/19	65.5 ± 6.4%	36/19	65.5 ± 6.4%	>0.999	<0.001

SMD: Standardized mean difference.

**Table 2:** Comparison of median overall survival with and without EHS in VP 3/4 group and in VP 0/1/2 group.

Median OS (m)	Extrahepatic Spread		*p*-Value
	Yes	No	
VP3/4 subgroup	13.0 (9.2–16.8)	15.0 (12.2–17.8)	0.407
VP ≤ 2 subgroup	11.4 (10.6–12.2)	19.4 (16.8–22.0)	<0.001

## Data Availability

The raw data supporting the conclusions of this article will be made available by the authors on request.
